# Foetal Movement Information and Maternal Concerns in the Third‐Trimester: Findings of an Aotearoa New Zealand National Survey

**DOI:** 10.1111/ajo.70122

**Published:** 2026-04-03

**Authors:** Billie F. Bradford, John M. D. Thompson, Christopher J. D. McKinlay, Likhit S. Dukkipati, George D. Cronin, Nimisha Waller, Judith McAra‐Couper, Tania Cornwall, Lesley M. E. McCowan, Robin S. Cronin

**Affiliations:** ^1^ Department of Obstetrics, Gynaecology and Reproductive Sciences University of Auckland Auckland New Zealand; ^2^ Department of Obstetrics and Gynaecology Monash University Melbourne Australia; ^3^ Department of Paediatrics: Child and Youth Health University of Auckland Auckland New Zealand; ^4^ Harmonic Analytics Wellington New Zealand; ^5^ Centre for Midwifery and Women's Health Research Auckland University of Technology Auckland New Zealand; ^6^ Sands Auckland Central New Zealand; ^7^ Women's Health Division Health New Zealand Te Whatu Ora Counties Manukau Auckland New Zealand

**Keywords:** antenatal care, foetal activity, foetal movement, pregnancy, pregnant women

## Abstract

**Background:**

Promoting awareness of foetal movement (FM) is a recognised approach to addressing preventable stillbirth. However, provision of information is inconsistent, and rates of presentation for decreased FM may vary by maternal factors. Information is lacking on maternal views of FM information and knowledge of actions if concerned about FM.

**Aims:**

To describe women's views of FM during late pregnancy in Aotearoa‐New Zealand (Aotearoa‐New Zealand), including factors that influence maternal actions if concerned.

**Materials and Methods:**

Online survey of women with singleton third‐trimester pregnancies in Aotearoa‐New Zealand. Multivariable analysis adjusted for maternal factors.

**Results:**

Eligible participants comprised European (1042, 63.5%), Māori (266, 16.2%), Pacific (119, 7.3%), Asian (160, 9.8%) and Other (35, 2.1%). Over half were nulliparous (916, 55.9%) and early third‐trimester (median 31, IQR 28–34). Most (1366, 83.3%) had been concerned about FM. Groups that were less likely to seek advice when concerned, included Māori (aOR 0.69, 95% CI 0.51%–0.93%, *p* = 0.02), Pacific (aOR 0.58, 95% CI 0.38%–0.88%, *p* = 0.01) and parous (aOR 0.73, 95% CI 0.59%–0.90%, *p* = 0.003) women. Advice seeking was also less likely with a doctor as the main maternity provider (aOR 0.59, 5% CI 0.39%–0.92%, *p* = 0.02) compared to a midwife, and those who received fewer than recommended antenatal visits (aOR 0.55, 95% CI 0.34%–0.88%, *p* = 0.01).

**Conclusions:**

This study identified that FM worries are common in the third‐trimester in Aotearoa‐New Zealand. Yet, some groups of women were less likely to seek advice when concerned, indicating opportunities to reduce inequity and address barriers to accessing care.

## 1 Introduction

Stillbirth rates are unacceptably high globally, with an estimated 1.9 million babies stillborn from 28 weeks' gestation each year [1]. Yet, rates vary significantly between countries, suggesting that lower stillbirth rates could be achieved, including in many high‐income countries [2]. Promoting awareness of foetal movements (FM) coupled with prompt evaluation when women present with decreased FM is recommended as a means of stillbirth prevention. Clinical practice guidelines typically advise, ‘All pregnant women should be routinely provided with written and verbal information about FM, including what to expect and when to contact the maternity provider’ [3]. However, studies suggest such information may not be consistently provided [4, 5], or when information is provided it may not be evidence‐based [6, 7].

Rates of presentation for decreased FM have been shown to vary according to maternal factors such as ethnicity, obesity and parity [8, 9]. In addition, psychological factors have been suggested to influence seeking care for decreased FM, although data are lacking. Although decreased frequency of FM is associated with stillbirth, some sources suggest perception of foetal hiccups or excessively strong FM should be considered a warning sign [10, 11], whereas others have reported perception of foetal hiccups and multiple episodes of unusually vigorous FM to be protective [12].

Stillbirth is associated with material disadvantage both between and within countries [2]. In high‐income countries, stillbirth rates are increased among women experiencing greater socio‐economic deprivation and non‐European ethnic minority groups [13]. Thus, approaches to stillbirth prevention should consider country‐specific sociodemographic factors when implementing and evaluating improvements. Consistent with international trends, stillbirth rates in Aotearoa‐New Zealand are higher among those living in areas of high deprivation and among women of Indian and Pacific ethnicity and under 20 years of age [14].

Little is known about maternal preferences for FM information provided in Aotearoa‐New Zealand and whether the advice received about decreased FM is evidence‐based and translated into practice. We performed an online survey to assess women's views of FM during late pregnancy, including factors that influence maternal actions if concerned.

## 2 Materials and Methods

The online survey was promoted on Facebook between 6 December 2021 and 28 February 2022. To support the participation of a diverse pregnant population, a research midwife provided face‐to‐face invitations to third‐trimester women attending antenatal clinics in Counties Manukau, an urban multi‐ethnic, low socio‐economic locality within Metropolitan Auckland. Eligibility criteria were 28 weeks' gestation or more, singleton pregnancy, living in Aotearoa‐New Zealand and completing the survey.

The protocol and survey tool were developed by RC and BB in consultation with a multidisciplinary team comprising researchers, research assistants and advisors of Māori and Indian ethnicity; bereaved parents and clinicians in neonatology, obstetrics and midwifery.

Data on potential confounding factors were collected at the time of participation in the survey and included maternal ethnicity (self‐defined and prioritised) [15], age, BMI (kg/m^2^) at pregnancy booking, education level, relationship status, urban/rural location and anxiety level [measured by the State Trait Anxiety Inventory (STAI)] [16]. Pregnancy characteristics comprised main maternity provider, parity, current gestation in weeks, adequacy of antenatal visits to date, hypertension, diabetes, currently smoking or vaping, going‐to‐sleep position, small‐for‐gestational‐age foetus, and foetal sex if known. We used the gestation of the first antenatal visit and number of antenatal visits to date to determine the adequacy of antenatal care (inadequate, care initiated ≥ 20 weeks' gestation or initiated < 20 weeks' with ≤ 80% of recommended antenatal visits; adequate, care initiated < 20 weeks' with > 80% of recommended visits) [17]. Categories with low frequency were combined. Imputation was undertaken for missing data: BMI was calculated using weight at survey if no booking weight (*n* = 21); STAI was calculated based on expert opinion of incomplete inventory (*n* = 7).

We conducted a multivariable analysis, using subject matter expertise to determine an initial set of explanatory variables while adjusting for confounding factors. Stepwise model selection, guided by the Akaike information criterion (AIC), was then used to refine the model by including or excluding variables. This method allowed us to identify the most parsimonious model that effectively explained the data with the fewest parameters. Statistical analyses were performed in R. The final best‐fit model retained only variables that significantly contributed to the variance in the response variable without any interaction effects.

Ethics approval was obtained from the Auckland Health Research Ethics Committee on 15/11/2021 (AH23362).

## 3 Results

**FIGURE 1:**
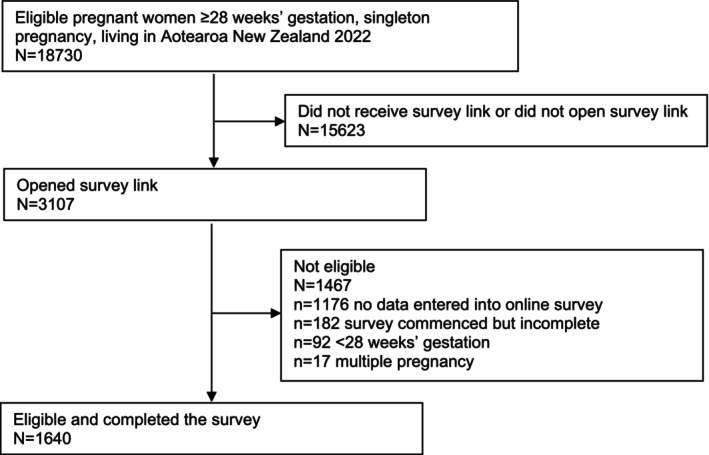
Flowchart of recruitment.

The survey was completed by 1640 eligible third‐trimester pregnant women living in Aotearoa‐New Zealand, approximately 3% of the total birthing population in 2022 (Figure 1).

The median (IQR) age was 31.0 years (28.0–34.0), booking BMI 26.0 kg/m^2^ (22.6–31.2) and gestation 32 weeks (29–35) (Table 1). More nulliparous women responded to the survey than parous (para 0, 55.9%, *n* = 916; para ≥ 1, 44.1%, *n* = 724) and more were in the early third‐trimester (28–32 weeks gestation, 55.7%, *n* = 914; 33–42 weeks, 44.3%, *n* = 726). Most (80.3%) had a community‐based midwife as their main maternity care provider, in keeping with the Aotearoa‐New Zealand maternity care system based on midwifery continuity‐of‐care. The majority had received adequate antenatal visits (71.1%, *n* = 1166). Ethnicities comprised 63.5% (*n* = 1042) European and 36.5% (*n* = 598) non‐European.

**TABLE 1:** Participant‐level characteristics at time of survey.

Demographic characteristics at time of survey	N = 1640 *n* (%) or median (IQR)
Ethnicity (in order of prioritisation)	
Māori	266 (16.2)
Pacific	119 (7.3)
Asian	178 (10.9)
Southeast/East Asian	98 (6.0)
South Asian	80 (4.9)
Other non‐European ethnicities	35 (2.1)
European	1042 (63.5)
Age in years	
Age median (IQR)	31.0 (28.0–34.0)
< 20	28 (1.7)
20–24	138 (8.4)
25–29	457 (27.9)
30–34	658 (40.1)
35–39	300 (18.3)
40+	59 (3.6)
BMI (kg/m^2^) at pregnancy booking	
BMI median (IQR)	26.0 (22.6–31.2)
< 18.5 (underweight)	33 (2.0)
18.5–24.9 (normal weight)	674 (41.1)
25–29.9 (overweight)	446 (27.2)
30–34.9 (obese class 1)	256 (15.6)
35–39.9 (obese class 2)	141 (8.6)
40+ (obese class 3)	90 (5.5)
Education level	
Primary education completed	216 (13.2)
Secondary education completed	146 (8.9)
Non‐university trade qualification	259 (15.8)
University degree	657 (40.1)
Post‐graduate degree	362 (22.1)
Relationship status	0
Single	90 (5.5)
Unmarried with partner	629 (38.4)
Married	921 (56.2)
Urban–rural location	
Urban	1117 (68.1)
Semi‐rural	303 (18.5)
Rural	220 (14.0)
State–Trait Anxiety Inventory	
Normal anxiety score	1358 (82.8)
High anxiety score	282 (17.2)
Pregnancy characteristics at time of survey	n (%) or median (IQR)
Main maternity provider	
Midwife community‐based	1317 (80.3)
Midwife hospital‐based	202 (12.3)
Private obstetrician	98 (6.0)
General Practitioner	23 (1.4)
Parity	
Nulliparous	916 (55.9)
Para 1	414 (25.2)
Para 2	194 (11.8)
Para 3+	116 (7.1)
Gestation	
Gestation weeks median (IQR)	32 (29–35)
28–32 weeks	914 (55.7)
33–36 weeks	490 (2.9)
37–39.9 weeks	206 (12.6)
40+ weeks	30 (1.8)
Antenatal visits	
Inadequate visits	474 (28.9)
Adequate visits	1166 (71.1)
Hypertension	
No	1541 (94.0)
Yes	99 (6.0)
Diabetes	
No	1515 (92.4)
Yes	125 (7.6)
Smoking/vaping	
No	1530 (93.3)
Yes smoke	58 (3.5)
Yes vape	52 (3.2)
Small‐for‐gestational‐age foetus	
No	1456 (88.8)
Yes	184 (11.2)
Sex of foetus	
Male	688 (42.0)
Female	648 (39.5)
Unknown	304 (18.5)

*Note:* Antenatal visits: inadequate, care initiated ≥ 20 weeks' gestation or initiated < 20 weeks' with < 80% of recommended visits; adequate, care initiated < 20 weeks' with ≥ 80% of recommended visits.

### 3.1 Foetal Movement Information

Nearly two‐thirds of respondents (62.8%, *n* = 1030) had received information about what FM to expect during pregnancy. However, 37.2% (*n* = 610) did not (File S1). When asked if they would like more FM information, over half (58.8%, *n* = 964) responded affirmatively; 44.4% (*n* = 728) wanted information about what FMs are normal, and 9.8% (*n* = 161) wanted to know what to do when worried about FM. One multiparous woman said, ‘I struggled with being asked if baby movements were normal and [my] midwife encouraged me to trust my body, but it's hard to know what normal is and how I could measure that’.

The preferred way to receive FM information was face‐to‐face (52.5%, *n* = 861), although some favoured other sources (including written information, 15.8%, *n* = 259; online, 14.0%, *n* = 229; phone apps, 9.6%, *n* = 158). When asked about the most trusted source of FM information, 72.4% (*n* = 1187) selected a midwife and 13.8% (*n* = 226) selected a doctor. One quarter (26.3%, *n* = 431) did not recall receiving advice about what to do if FM decreased.

We considered what factors may influence receiving advice on what to do if FM decreases. Following multivariable analysis, we found women were significantly less likely to receive decreased FM advice if they were of Māori (0.72, 95% Confidence Interval [CI] 0.53–0.99) or Southeast/East Asian ethnicity (0.61, 95% CI 0.38–0.96), < 20 years of age (0.30, 95% CI 0.13–0.70), or parity ≥ 1 (0.74, 0.58–0.99) (Table 2).

**TABLE 2:** Maternal factors and receiving advice about what to do if fetal movements decrease.

Maternal/pregnancy characteristics	*N* = 1640	Univariable Odds Ratio (95% CI)	Adjusted Odds Ratio (95% CI)	*p*
Ethnicity (prioritised)				
European & other	1077	1	1	
Māori	266	0.66 (0.49–0.88)	0.72 (0.53–0.99)	0.04
Pacific	119	0.89 (0.58–1.36)	0.94 (0.59–1.49)	0.79
Southeast/East Asian	98	0.64 (0.41–0.99)	0.61 (0.38–0.96)	0.03
Indian	80	1.54 (0.85–2.78)	1.31 (0.71–2.41)	0.38
Parity				
Para 0 (nulliparous)	916	1	1	
Para ≥ 1 (parous)	724	0.81 (0.65–1.01)	0.74 (0.58–0.95)	0.01
Age in years				
< 20	28	0.40 (0.18–0.85)	0.30 (0.13–0.70)	0.01
20–24	138	0.94 (0.62–1.42)	0.86 (0.55–1.34)	0.50
25–29	457	0.96 (0.73–1.26)	0.92 (0.69–1.22)	0.54
30–34	658	1	1	
35–39	300	1.07 (0.78–1.46)	1.16 (0.83–1.62)	0.38
≥ 40	59	0.62 (0.35–1.09)	0.67 (0.37–1.20)	0.17
Main maternity provider				
Midwife	1519	1	1	
Doctor	121	0.57 (0.38–0.87)	0.59 (0.38–0.92)	0.02
Gestation in weeks				
28–32	914	1	1	
33–36	490	2.45 (1.87–3.21)	2.40 (1.82–3.17)	< 0.001
37–40+	236	2.76 (1.89–4.10)	2.78 (1.88–4.10)	< 0.001
Antenatal visits				
Adequate	1166	1	1	
Inadequate	474	0.59 (0.38–0.92)	0.55 (0.34–0.90)	0.02
Small for gestational age foetus				
No	1458	1	1	
Yes	184	1.88 (1.25–2.81)	1.66 (1.09–2.52)	0.02

*Note:* Variables for selection in the model prior to stepwise regression: ethnicity, parity, age in years, BMI (kg/m^2^), education level, relationship status, urban/rural location, anxiety level, main maternity provider, parity, gestation weeks, adequacy of antenatal visits, hypertension, diabetes, currently smoking or vaping, small for gestational age foetus.

Other factors associated with lower odds of receiving information on what to do if FM decreases included having a doctor as the main maternity provider (aOR 0.59, 95% CI 0.38–0.92, *p* = 0.02) and receiving inadequate antenatal visits (aOR 0.55, 95% CI 0.34–0.90, *p* = 0.02). Reassuringly, women were more likely to have received decreased FM information if their foetus was diagnosed as small‐for‐gestational‐age (aOR 1.66, 95% CI 1.09–2.52, *p* = 0.02) or if they were 33–36 weeks' (aOR 2.40, 95% CI 1.82–3.17, *p* ≤ 0.001) or 37–40+ weeks' (aOR 2.78, 95% CI 1.88–4.10, *p* ≤ 0.001) gestation (Table 2).

### 3.2 Foetal Movement Concerns

We asked participants about FM concerns during this pregnancy. Most (83.3%, *n* = 1366) had been concerned about FM at least once, with just 16.7% (*n* = 274) indicating that they had never worried (File S2).

When asked who the first person they talked to when concerned about FM was, 58.3% (796 of 1366 with FM concerns) spoke to their partner or another family/whānau member first. This woman explained, ‘I was worried at one stage, but just discussed it with my husband and then left it alone, baby started kicking again a few hours later’. Just 14.5% (*n* = 199) reported that they would talk to a doctor or midwife first.

Over half (56.6%, 773 of 1366 with FM worries) sought advice from a maternity provider when concerned about FM. Of those who had not (43.4%, *n* = 593), the main reasons were that FM returned to normal (33.1%, *n* = 452) or that they had not worried enough to seek advice (28.7%, *n* = 392). Concerningly, some women received incorrectly reassuring information from family/whānau/friends and social media/internet sources (8.6%, *n* = 117) or wished to avoid bothering the maternity provider (8.1%, *n* = 111). One woman said, ‘I didn't want to bother the midwife, and I didn't want to know something was wrong as my anxiety can skyrocket with these sorts of things’.

We asked participants how comfortable they would feel contacting a doctor or midwife when they had FM concerns. Comfort was generally high, with 83.8% (*n* = 1375) feeling moderately to very comfortable. Travelling to assess FM concerns could be challenging, with 12.8% (*n* = 210) reporting that travel was not easy or only somewhat easy, mainly due to distance from a hospital and lack of childcare. When adjusted for other factors in a multivariable analysis, only a high anxiety level was significantly and negatively associated (aOR 0.38, 95% CI 0.22–0.68, *p* < 0.001) with comfort about contacting a doctor or midwife (File S3). One participant explained, ‘I would be comfortable if I seriously thought something was wrong/had changed, but I'd feel like a hypochondriac if wasn't sure’.

In multivariable analysis, seeking maternity provider advice when concerned about FM was associated with ethnicity and parity, with lower odds of seeking advice for Māori (aOR 0.69, 95% CI 0.51–0.93, *p* = 0.02), Pacific (aOR 0.58, 95% CI 0.38–0.88, *p* = 0.01) and parous (aOR 0.73, 95% CI 0.59–0.90, *p* = 0.003) women. Women also had lower odds of seeking advice if a doctor was their main maternity provider (aOR 0.59, 5% CI 0.39–0.92, *p* = 0.02) compared to a midwife, and if they had inadequate antenatal visits (aOR 0.55, 95% CI 0.34–0.88, *p* = 0.01) (Table 3).

**TABLE 3:** Factors associated with seeking care when concerned about foetal movements.

Factors	*N* = 1640	Univariable odds ratio (95% CI)	Adjusted odds ratio (95% CI)	*p*
Ethnicity (prioritised)				
European	1077	1	1	
Māori	266	0.84 (0.64–1.10)	0.69 (0.51–0.93)	0.02
Pacific	119	0.67 (0.45–0.98)	0.58 (0.38–0.88)	0.01
Southeast/East Asian	98	1.15 (0.76–1.73)	1.25 (0.81–1.92)	0.31
South Asian	80	1.29 (0.82–2.04)	1.28 (0.80–2.07)	0.31
Parity				
Para 0	916	1	1	
Para ≥ 1	724	0.75 (0.61–0.91)	0.73 (0.59–0.90)	0.003
Education level				
University	1019	1	1	
Non‐university	621	1.25 (1.02–1.53)	1.27 (1.01–1.60)	0.04
Relationship status				
Married	921	1	1	
Unmarried with partner	629	1.14 (0.93–1.39)	1.05 (0.84–1.32)	0.65
Single	90	1.69 (1.09–2.63)	1.66 (1.02–2.69)	0.04
State–Trait Anxiety Inventory				
Normal anxiety score	1351	1	1	
High anxiety score	282	1.62 (1.26–2.11)	1.50 (1.15–1.97)	0.003
Main maternity provider				
Midwife	1519	1	1	
Doctor	121	0.56 (0.37–0.86)	0.59 (0.39–0.92)	0.02
Gestation in weeks				
28–32	914	1	1	
33–36	490	1.24 (1.00–1.55)	1.18 (0.94–1.48)	0.16
37–40+	236	1.94 (1.45–2.60)	1.83 (1.35–2.47)	< 0.001
Antenatal visits				
Adequate	1166	1	1	
Inadequate	474	0.60 (0.38–0.93)	0.55 (0.34–0.88)	0.01
Smoke or vape				
No	1530	1	1	
Yes	110	2.01 (1.34–3.00)	1.98 (1.27–3.09)	0.003
Small for Gestational Age foetus				
No	1458	1	1	
Yes	184	2.02 (1.47–2.77)	1.80 (1.30–2.51)	< 0.001

*Note:* Variables for selection in the model prior to stepwise regression: ethnicity, parity, age in years, BMI (kg/m^2^), education level, relationship status, urban/rural location, anxiety level, main maternity provider, gestation weeks, adequacy of antenatal visits, hypertension, diabetes, currently smoking or vaping, small for gestational age foetus.

In addition, respondents were over 1.5 times more likely to seek advice from a doctor or midwife when worried about FM if they were 37–40+ weeks' pregnant (aOR 1.83, 95% CI 1.35–2.47, *p* < 0.001), smoked or vaped (aOR 1.98, 95% CI 1.27–3.09, *p* = 0.003), had a single relationship status (aOR 1.66, 95% CI 1.02–2.69, *p* = 0.04), had a high anxiety score (aOR 1.50, 95% CI 1.15–1.97, *p* = 0.003) or had been advised that their baby was small for gestational age (aOR 1.80, 95% CI 1.30–2.51, *p* < 0.001). Those without a university‐level education were also more likely to seek advice (aOR 1.27, 95% CI 1.01–1.60, *p* = 0.04).

## 4 Discussion

In this survey of 1644 singleton third‐trimester pregnant women in Aotearoa‐New Zealand, we described women's views of FM, including factors that influenced maternal actions when concerned. FM concerns were commonplace, yet nearly half of the pregnant participants did not contact a maternity provider when they were worried. Some groups of women were less likely to seek advice for FM concerns, indicating opportunities to reduce inequity and address barriers to accessing care.

Notably, a survey of women receiving antenatal care in Auckland, Aotearoa‐New Zealand, in 2012 [18], reported that 62% had received information about what FM to expect, virtually the same proportion as the 63% reported in the current study. Thus, despite a general increase in guidance for maternity providers about FM awareness, and an increase in women seen in a hospital for FM concerns [19], there appears to have been little improvement in information sharing about FM expectations over the past decade. However, it was encouraging to find an increase in the proportion of women recalling advice about what to do if FM decreases (74% in the current study vs. 40% in 2012).

Clinical practice guidelines recommend women be given information about FM by 28 weeks' gestation. Furthermore, an international individual participant data analysis of stillbirth case–control studies [12] found that the association of late stillbirth with the perception of decreased frequency of FM is stronger in the early third‐trimester (28–32 weeks' gestation) than later in pregnancy. Therefore, it was suboptimal that our early third‐trimester participants were less likely to have received FM information and offers an important reminder that pregnant women should not receive this information too late.

Concerningly, some groups with higher rates of perinatal death in Aotearoa‐New Zealand [14]—Māori, Pacific, and teenage (< 20 years of age) mothers—had lower odds of contacting a maternity provider when worried about FM. Those with a doctor as their main maternity provider, parous women, or who reported inadequate antenatal visits were also less likely to seek care. Previous studies have demonstrated a relationship between receiving FM awareness information and care‐seeking when concerned [20, 21]. It is possible that the lower rates of contacting a maternity provider with FM worries are related to the shortage of continuity‐of‐care midwives, which disproportionately affects Māori, Pacific, teenage and parous mothers [22, 23] and in turn is associated with inadequate antenatal care.

Some maternal worry about FM can be considered a normal part of pregnancy due to increasing attachment to the baby and greater maternal attention to FM. Instances of worry, where 42% of women did not seek maternity provider care, were primarily attributed to not being worried enough (29%) or that FM returned to normal (33%), suggesting that in most instances, the concerns were mild or self‐limiting. The first person the majority (58%) of women spoke to about their worry was their partner or other family/whānau. This illustrates the importance of including the partner and family/whānau in FM information sharing. It also suggests that when women do contact a maternity provider about FM concerns, their degree of worry is likely to be high or reflect a longer period of decreased FM.

Women with high anxiety scores were more likely to contact a maternity provider with FM concerns. Maternal psychological states can interact with foetal activity [24], whereas maternal stress is suggested to have potentially negative effects on foetal development [25, 26]. Regardless of the nature of the relationship between maternal anxiety and presentation for decreased FM, we found that women with anxiety were also less comfortable contacting their maternity provider when concerned. This serves as a reminder for maternity providers to respond compassionately to such presentations.

Women with a known small‐for‐gestational‐age foetus and those who smoked or vaped were less likely to report that FM was easy to feel. This is in keeping with other reports [27]. However, it was reassuring that these women were both more likely to receive information about FM and more likely to present when worried about decreased FM.

A strength of this study is the large multiethnic sample and detailed analysis of maternal characteristics. Although we did not have access to maternity records and pregnancy outcome information, many findings agree with a cross‐sectional study of third‐trimester women with subsequent normal pregnancy outcomes [28]. A limitation is that participants were largely in the early third‐trimester and nulliparous. This may reflect a greater interest in FM for first‐time mothers, particularly in the early third‐trimester where FM reaches the greatest frequency. However, the sizeable number of participants and multivariable analysis have allowed us to examine differences according to important sub‐groups within the Aotearoa‐New Zealand population.

In summary, we found that FM worries were normal. While most were comfortable seeking advice from a maternity provider with FM concerns, inequities were identified for some groups. Women at increased risk of adverse perinatal outcomes (including Māori, Pacific, and teenage mothers, and those who received inadequate antenatal visits) were less likely to have received FM advice and to contact a maternity provider when worried. It is important that health services address these barriers to equitable maternity outcomes by ensuring that information about what action to take when worried about decreased FM is shared with all women in a timely manner.

## Funding

This research was supported by Health NZ‐Te Whatu Ora Counties Manukau Ko Awatea Tupu Research Fund and a Summer Studentship from the Auckland University of Technology. The views expressed are those of the authors and not the funders.

## Ethics Statement

The Research Ethics Committee provided approval on 15 November 2021. Participants consented for their anonymised data to be published as part of the informed consent process. Data are not publicly available due to the potentially identifiable nature of interview transcripts but will be made available upon reasonable request to the corresponding author.

## Conflicts of Interest

The authors declare no conflicts of interest.

## Supporting information


**File S1:** Advice on foetal movements.
**File S2:** Foetal movement concerns this pregnancy.
**File S3:** Comfort contacting maternity provider with foetal movement concerns.

## Data Availability

The data that support the findings of this study are available from the corresponding author upon reasonable request.
