# Supplementary material for: Foetal Movement Information and Maternal Concerns in the Third‐Trimester: Findings of an Aotearoa New Zealand National Survey
Source: Aust N Z J Obstet Gynaecol. 2026 Apr 3;66(2):e70122. doi: 10.1111/ajo.70122 (PMC13049267; doi:10.1111/ajo.70122)
Supplement: Supplementary file 1 — File S1: Advice on foetal movements. File S2: Foetal movement concerns this pregnancy. File S3: Comfort contacting maternity provider with foetal movement concerns. [file AJO-66-0-s001.docx]

**Supplementary files**

| Supplementary File 1: Advice on fetal movements | |
| --- | --- |
|  | N=1640  n (%) |
| Received maternity provider advice on FM to expect |  |
| Yes | 1030 (62.8) |
| No | 610 (37.2) |
| More FM advice desired |  |
| No - have sufficient | 676 (41.2) |
| Yes - what FM are normal | 728 (44.4) |
| Yes - what to do if worried about FM | 161 (9.8) |
| Yes - where to get more FM advice/information | 75 (4.6) |
| Preferred way to receive FM advice |  |
| Face-to-face with maternity provider | 861 (52.5) |
| Written information (pamphlets, books etc) | 259 (15.8) |
| Online (pregnancy websites, online forums etc) | 229 (14.0) |
| Mobile phone applications | 158 (9.6) |
| Talking on the phone with health professional | 61 (3.7) |
| Social media (Instagram, Facebook, TikTok etc) | 43 (2.7) |
| Video call with health professional | 16 (1.0) |
| Email from health professional | 13 (0.8) |
| Must trusted source of FM advice |  |
| Midwife | 1187 (72.4) |
| Doctor | 226 (13.8) |
| Internet | 50 (3.0) |
| Childbirth educator | 43 (2.6) |
| Family/whānau and friends | 37 (2.3) |
| Written information (pamphlets, books etc) | 26 (1.6) |
| Health professional other than midwife/doctor | 19 (1.2) |
| Self-perception | 16 (1.0) |
| Research articles | 12 (0.7) |
| Phone application | 12 (0.7) |
| Do not trust any source of information | 12 (0.7) |
| Received maternity provider advice on what to do if FM decrease | |
| Yes | 1209 (73.7) |
| No | 431 (26.3) |

FM=fetal movements

| Supplementary File 2: Fetal movement concerns this pregnancy | |
| --- | --- |
|  | N=1640 |
|  | n (%) |
| Frequency of concerns about FM |  |
| Never worried | 274 (16.7) |
| Once | 508 (31.0) |
| More than once but rarely | 513 (31.3) |
| Sometimes | 260 (15.9) |
| Often | 73 (4.5) |
| Always worried | 12 (0.7) |
| First person you talked to when concerned |  |
| Partner | 721 (44.0) |
| Not applicable, never worried about FM | 274 (16.7) |
| No-one, as not worried enough | 254 (15.5) |
| Midwife | 187 (11.4) |
| Worried, but didn’t talk to anyone | 67 (4.1) |
| Mother | 59 (3.6) |
| Friend | 42 (2.6) |
| Other family/whānau member | 16 (1.0) |
| Doctor | 12 (0.7) |
| Comfort contacting maternity provider if any FM concerns |  |
| Not comfortable making contact | 55 (3.4) |
| Somewhat comfortable making contact | 210 (12.8) |
| Moderately comfortable making contact | 373 (22.7) |
| Very comfortable making contact | 1002 (61.1) |
| Ease of travel to maternity provider for assessment if any FM concerns |  |
| Travel not easy | 48 (2.9) |
| Travel somewhat easy | 162 (9.9) |
| Travel moderately easy | 354 (21.6) |
| Travel very easy | 1076 (65.6) |
| Contacted maternity provider when concerned |  |
| Worried, contact not attempted | 593 (36.2) |
| Worried, contact made | 767 (46.8) |
| Not applicable, never worried | 274 (16.7) |
| Worried, contact unsuccessful | 6 (0.4) |
| Main reason for not contacting maternity provider when concerned |  |
| FM returned to normal | 452 (27.6) |
| Not worried enough to contact maternity provider | 392 (23.9) |
| Not applicable, never worried about FM | 274 (16.7) |
| Not applicable, contact made with maternity provider | 183 (11.2) |
| Didn’t want to bother maternity provider | 111 (6.8) |
| Advised not to worry by family/whānau/friends | 110 (6.7) |
| Decided to wait until next pregnancy appointment | 48 (2.9) |
| Thought baby was sleeping | 35 (2.1) |
| Didn’t want to go to hospital | 10 (0.6) |
| Advised not to worry by social media/internet | 7 (0.4) |
| Thought it was normal for FM to slow near birth | 7 (0.4) |
| Worried, contact unsuccessful | 6 (0.4) |
| Didn’t want to know if something was wrong | 5 (0.3) |

FM=fetal movements

| Supplementary File 3: Comfort contacting maternity provider with fetal movement concerns | | | | |
| --- | --- | --- | --- | --- |
|  | **N=1640** | **Univariable Odds Ratio (95% CI)** | **Adjusted Odds Ratio (95% CI)** | **P value** |
| State-Trait Anxiety Inventory |  |  |  |  |
| Normal anxiety score | 1351 | 1 | 1 |  |
| High anxiety score | 282 | 0.38 (0.21-0.67) | 0.38 (0.22-0.68) | **<0.001** |

Variables for selection in the model prior to stepwise regression: ethnicity, parity, age in years, BMI (kg/m2), education level, relationship status, urban/rural location, anxiety level, main maternity provider, parity, gestation weeks, adequacy of antenatal visits, hypertension, diabetes, currently smoking or vaping, small for gestational age fetus.
